# Ino2, activator of yeast phospholipid biosynthetic genes, interacts with basal transcription factors TFIIA and Bdf1

**DOI:** 10.1007/s00294-023-01277-z

**Published:** 2023-11-10

**Authors:** Maike Engelhardt, Stefan Hintze, Eva-Carina Wendegatz, Julia Lettow, Hans-Joachim Schüller

**Affiliations:** 1 Center for Functional Genomics of Microbes, Institut für Genetik und Funktionelle Genomforschung, Universität Greifswald, Felix-Hausdorff-Strasse 8, 17487 Greifswald, Germany; 2 Present Address: Cheplapharm, Greifswald, Germany; 3 Present Address: Friedrich-Baur-Institut an der Neurologischen Klinik und Poliklinik, LMU Klinikum, Munich, Germany

**Keywords:** *Saccharomyces cerevisiae*, Coactivator, TFIIA, Toa1, Bdf1, Activator-binding domain

## Abstract

Binding of general transcription factors TFIID and TFIIA to basal promoters is rate-limiting for transcriptional initiation of eukaryotic protein-coding genes. Consequently, activator proteins interacting with subunits of TFIID and/or TFIIA can drastically increase the rate of initiation events. Yeast transcriptional activator Ino2 interacts with several Taf subunits of TFIID, among them the multifunctional Taf1 protein. In contrast to mammalian Taf1, yeast Taf1 lacks bromodomains which are instead encoded by separate proteins Bdf1 and Bdf2. In this work, we show that Bdf1 not only binds to acetylated histone H4 but can also be recruited by Ino2 and unrelated activators such as Gal4, Rap1, Leu3 and Flo8. An activator-binding domain was mapped in the N-terminus of Bdf1. Subunits Toa1 and Toa2 of yeast TFIIA directly contact sequences of basal promoters and TFIID subunit TBP but may also mediate the influence of activators. Indeed, Ino2 efficiently binds to two separate structural domains of Toa1, specifically with its N-terminal four-helix bundle structure required for dimerization with Toa2 and its C-terminal β-barrel domain contacting TBP and sequences of the TATA element. These findings complete the functional analysis of yeast general transcription factors Bdf1 and Toa1 and identify them as targets of activator proteins.

**Supplementary Information:**

The online version contains supplementary material available at 10.1007/s00294-023-01277-z.

## Introduction

Recruitment of general transcription factor TFIID to basal promoters of eukaryotic structural genes is an essential and rate-limiting step for subsequent initiation of transcription by RNA polymerase II (reviewed by Hahn and Young 2011; Warfield et al. 2017) which is supported by TFIIA at certain control regions (Lieberman et al. 1997; Papai et al. 2010). Consequently, TFIID containing the TATA-binding protein TBP and (in yeast) 14 TBP-associated factors (Tafs) not only contacts DNA sequence elements of basal promoters such as the TATA box and initiator (Louder et al. 2016) but also interacts with transcriptional activators such as Rap1 (Garbett et al. 2007) which thereby facilitate promoter occupation by TFIID and support formation of the pre-initiation complex (PIC). Especially, TFIID subunit Taf1 has been described as a “versatile transcriptional toolbox” (Wassarman and Sauer 2001) and executes a number of distinct functions. Taf1 contains “winged helix” (WH) and “zinc knuckle” structural motifs (Curran et al. 2018) which are presumably involved in binding to mammalian basal promoter elements. Taf1 N-terminal domain (TAND) negatively regulates TBP binding to the TATA element which may be overcome by interaction of activator proteins with TAND (Anandapadamanaban et al. 2013). However, the existence of a Taf1 histone acetyltransferase domain has been questioned by analyzing structural data of a Taf1–Taf7 subcomplex (Bhattacharya et al. 2014). We have previously shown that the yeast transcriptional activator Ino2 of phospholipid biosynthetic genes is able to interact with two separate subdomains of Taf1 (aa 1–100 = ABD1, overlapping with TAND; aa 180–250 = ABD2) and that Taf1 requires Ino2 for recruitment to target genes (Hintze et al. 2017). Structural studies revealed that nine Taf proteins contain histone fold domains (Selleck et al. 2001) indicating that heterodimeric sub-complexes within TFIID may mimic a nucleosome-related organization of promoter DNA. Interestingly, four of these Taf proteins (Taf4, Taf6, Taf10 and Taf12) could bind to Ino2 and other activators such as Rap1 and Gal4 (Hintze et al. 2017; Layer and Weil 2013; Reeves and Hahn 2005). Importantly, human Taf1 contains two carboxy-terminal bromodomains which can bind to acetylated histone H4 (Jacobson et al. 2000), while these domains are absent from yeast Taf1. Instead, the missing part of yeast Taf1 is found within individual protein Bdf1 (bromodomain factor 1) or the related Bdf2 both of which interact with Taf7 and thus functionally complete the TFIID complex, including a protein kinase activity which has been mapped to the carboxy-terminal sequences of Bdf1 and Bdf2 (Matangkasombut et al. 2000). Bdf1 can interact with the N-terminus of histone H4 (Pamblanco et al. 2001) and has been also identified as a subunit of the chromatin remodeling complex SWR1 responsible for the exchange of conventional histone H2A against the variant H2A.Z (Nguyen et al. 2013). A comprehensive RNA-seq study with a *bdf2* deletion strain also containing a degron-coupled Bdf1 variant showed that depletion of Bdf proteins caused transcriptional changes even more distinctive than depletion of TFIID subunits (Donczew and Hahn 2021). As a phenotypic example, loss of Bdf1 renders yeast cells sensitive against osmotic stress, while overexpression of *BDF2* could suppress salt sensitivity of a *bdf1* mutant (Liu et al. 2007). Interestingly, acetylation of histone H4 by NuA4 is not essentially required for promoter targeting of Bdf1 (Donczew and Hahn 2021).

General transcription factor TFIIA was initially described as being important for in vitro transcription under some (but not all) conditions, depending on the purity of the test system. TFIIA of *S. cerevisiae* is a heterodimeric complex of subunits encoded by essential genes *TOA1* and *TOA2* (Ranish et al. 1992), while human TFIIA contains three subunits α, β (processed from a larger precursor and together corresponding to Toa1; DeJong and Roeder 1993), and γ (related to Toa2; Sun et al. 1994). For TFIIA, three separate functions have been described: (1) TFIIA directly contacts TBP (Geiger et al. 1996; Tan et al. 1996), thereby stabilizing the binding of TBP to the TATA box (Imbalzano et al. 1994; Weideman et al. 1997; Cianfrocco et al. 2013) and stimulating the interaction of TFIID with basal promoter sequences, especially by preventing formation of TFIID dimers (Coleman et al. 1999). Interaction of yeast TFIIA with TBP is stimulated by phosphorylation of its Toa1 subunit, possibly by casein kinase II and/or by Taf1/Bdf (Solow et al. 1999; 2001). The positive influence of TFIIA on TBP–TATA binding was shown to be particularly effective with a non-consensus TATA sequence (Stewart and Stargell 2001; Hieb et al. 2007). In the yeast *Schizosaccharomyces pombe*, TFIIA + TBP (but not TBP alone) has been recently shown to bind to the initiator sequence of the strongly regulated *nmt1* promoter (Rojas et al. 2022). Human TFIIA not only supports TBP binding to the basal promoter but also interacts with a G-rich sequence motif closely upstream of the TATA element (designated IIARE, overlapping with the TFIIB recognition element, BRE^u^; Wang et al. 2017). Contacts of 3–5 bases upstream of the TATA motif have been also shown for yeast TFIIA by X-ray structural analyses (Geiger et al. 1996; Tan et al. 1996). TFIID, TFIIA and TFIIB together with promoter DNA form the upstream promoter complex (Sainsbury et al. 2015). (2) TFIIA also functions as a coactivator, mediated by contacts to several activator proteins such as yeast Gal4 and Rap1, mammalian AP-1 and viral VP16 (Kobayashi et al. 1995; Ozer et al. 1996; Stargell et al. 2000; Layer and Weil 2013). The coactivator function of TFIIA is further supported by the finding that increased gene dosage of *TOA1* and *TOA2* can suppress activation defects caused by mutant yeast TBP (Liu et al. 1999). (3) TFIIA counteracts negative regulators of TBP such as the heterodimeric NC2 (= Dr1/DRAP) which prevents interaction of TBP with TFIIB and subsequent formation of the pre-initiation complex (Meisterernst and Roeder 1991). Genetic and biochemical studies support the hypothesis that TFIIA and NC2 compete for interaction with TBP, thereby influencing the equilibrium between transcriptional activation and repression (Xie et al. 2000). In addition to TFIID and TFIIA, RNA polymerase II holoenzyme (comprising core RNA polymerase II, mediator and general factors TFIIB, TFIIF and TFIIH) together with TFIIE is required for finally initiating transcription and forming an open complex with unpaired DNA (Koleske and Young 1995). This pre-initiation complex responds to transcriptional regulators which may also contact TFIIB and subunits of mediator (Koleske and Young 1995).

In this work, we investigated whether *S. cerevisiae* bromodomain proteins Bdf1 and Bdf2 (which are synthesized separately from the Taf1 subunit of TFIID) and basal transcription factor TFIIA can interact with transcriptional activation domains of activator Ino2. We could demonstrate that Bdf1 (but not Bdf2) is able to contact TAD2 of Ino2 (but not TAD1), while subunit Toa1 of TFIIA interacts with TAD1 and TAD2. Using length variants of Bdf1 and Toa1 we also could define minimal activator-binding domains (ABDs), demonstrating that conserved domains of Toa1 at its N- and C-terminus, respectively, mediates binding to activators.

## Materials and methods

### Strains, media, and growth conditions

Yeast protein extracts utilized for in vitro interaction assays were prepared from transformants of *S. cerevisiae* strain C13-ABY.S86 deficient for vacuolar proteinases (*pra1 prb1 prc1 cps1*; De Antoni and Gallwitz 2000). Synthetic media for selection of transformants and the procedure of yeast transformation has been described (Schwank et al. 1995). To prepare bacterial protein extracts, *E. coli* strain BL21-CodonPlus(DE3)-RP (Stratagene/Agilent) containing additional tRNA genes was used. Genotypes of *S. cerevisiae* strains used in this work are shown in Table S1 (Supplementary material).

### Plasmid constructions

To subsequently perform in vitro interaction studies, the *E. coli* expression plasmid pGEX-2TK encoding glutathione-*S*-transferase (GST) under control of the IPTG-inducible tac promoter-operator was used to construct GST fusions with transcriptional activation domains of Ino2, Rap1, Gal4, Aro80, Leu3, Swi5 and Flo8. *GST–INO2* fusions have been described (Hintze et al. 2017), while TAD-encoding sequences were amplified by PCR using oligonucleotides specific for *RAP1*, *GAL4*, *ARO80*, *LEU3*, *SWI5* and *FLO8*, respectively.

HA_3_-fusion proteins were prepared from either *S. cerevisiae* or *E. coli*, using expression plasmids derived from p426-MET25HA (*MET25* promoter, inducible by the absence of methionine; Mumberg et al. 1994) or pASK-IBA5-HA3 (tet promoter-operator, inducible with 0.2 mg/L anhydrotetracycline; IBA, Göttingen, Germany).

To introduce missense mutations into the coding regions of *BDF1* and *TOA1*, the QuikChange site-directed mutagenesis kit (Agilent) in combination with pairs of mutagenic primers replacing selected natural codons against an alanine-specific codon was used. The authenticity of *BDF1* and *TOA1* mutational variants was verified by DNA sequencing (LGC Genomics, Berlin, Germany). Genotypes of expression plasmids and gene-specific primers used for their construction are shown in Tables S2 and S3, respectively (Supplementary material).

### In vitro interaction assays

In vitro interaction assays were essentially performed as described by Wagner et al. (2001). Total protein extracts from yeast transformants were prepared by mechanical agitation in the presence of zirconia beads and from *E. coli* transformants by sonication. To ensure that similar amounts of GST fusions are used as “bait” proteins, GST enzyme assays were performed. GST fusions were then immobilized on glutathione (GSH) sepharose beads and incubated with total protein extracts from yeast or *E. coli* containing HA fusions of Bdf1, Bdf2, Toa1 or Toa2. Prior to elution with free GSH, beads were washed twice with buffer A1. Eluted proteins were separated by SDS/PAGE, transferred to a PVDF membrane and incubated with anti-HA-peroxidase conjugate (monoclonal antibody 12CA5 conjugate; Sigma-Aldrich). Visualization of antibody-bound HA-tagged proteins was achieved by treatment with a POD chemiluminescent substrate and subsequent detection of the luminescence with a digital imager (ChemoStar, Intas).

### Plasmid shuffling

Functional analysis of *TOA1* variants was performed using the plasmid shuffling strategy described by Sikorski and Boeke (1991). *TOA1* together with its native promoter and terminator was PCR amplified and inserted into *ARS CEN URA3* vector pRS416 (Agilent/Stratagene). The resulting rescue plasmid pMS114 was transformed into wild-type strain JS91.15–23 (Schwank et al. 1995). Next, the entire coding region of the chromosomal *TOA1* gene was deleted, using a *toa1*Δ*::LEU2* gene disruption cassette (plasmid pMS113). For future use of *LEU2* as a selection marker, Cre recombinase was induced to remove *LEU2* flanked by loxP sites from the disruption cassette (Güldener et al. 1996) to finally give strain MSY8. *ARS CEN LEU2 TOA1* plasmid pMS115 derived from YCplac111 (Gietz and Sugino 1988) was then used to construct 11 different missense variants by site-directed mutagenesis. The desired mutations within *TOA1* and the absence of unwanted alterations were verified by DNA sequencing. Incubation of transformants on synthetic medium containing 5-fluoroorotic acid (FOA) allowed counter-selection against pMS114 and phenotypic characterization of the resulting strains in the absence or presence of inositol + choline.

## Results

### Bdf1 interacts with a single TAD of Ino2

**Fig. 1:**
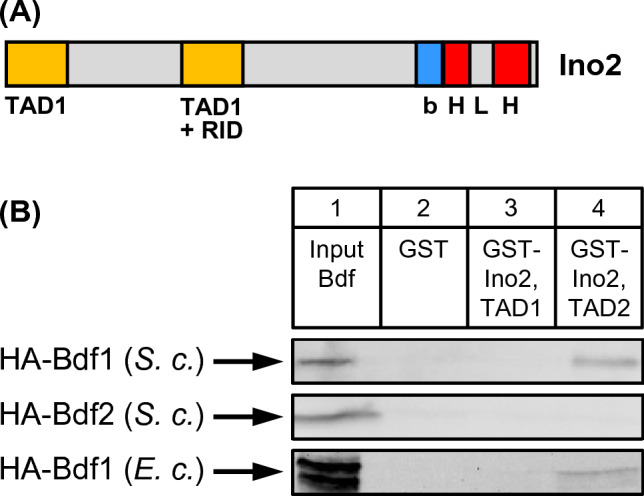
**A** Functional domains of transcriptional activator Ino2. bHLH: basic helix–loop–helix domain for heterodimerization with Ino4 and binding to activating sequences of phospholipid biosynthetic genes; TAD1, TAD2: transcriptional activation domains; RID: repressor interaction domain which is bound by Opi1. **B** In vitro interaction assays (GST pull-down) with Ino2 activation domains and Bdf proteins. Fusion proteins GST–Ino2 TAD1 (aa 1–35; encoded by pSH117) and GST–Ino2 TAD2 (aa 101–135; pSH118) were synthesized in *E. coli*, immobilized on GSH sepharose and incubated with protein extracts containing epitope-tagged Bdf1 (full-length; synthesized by yeast expression plasmid pJL84 and *E. coli* expression plasmid pMS161, respectively) or Bdf2 (full-length; synthesized by yeast expression plasmid pJL85). Negative control experiments were performed with GST (pGEX-2TK)

We have previously shown that both activation domains TAD1 and TAD2 of Ino2 (cf. Figure 1A for functional domains of Ino2) are able to interact with several subunits of basal transcription factor TFIID (Hintze et al. 2017). In contrast to mammalian cells, yeast TFIID subunit Taf1 is devoid of C-terminal bromodomains which can bind to acetylated histone H4 and presumably facilitate access of TFIID to chromatin. Instead, the missing bromodomains are provided by individual proteins Bdf1 and Bdf2 which are associated with TFIID and have been shown to be part of the transcriptional pre-initiation complex (Matangkasombut et al. 2000). To investigate whether Bdf1 and Bdf2 are also bound by Ino2, we constructed yeast expression plasmids encoding full-length variants HA-Bdf1 and HA-Bdf2 and used them for preparation of protein extracts from corresponding transformants. For GST pull-down interaction experiments, GST fusions of Ino2 TAD1 (aa 1–35) and Ino2 TAD2 (aa 101–135) were immobilized on GSH sepharose. As is shown in Fig. 1, Bdf1 (but not Bdf2) synthesized in *S.* *cerevisiae* could interact with activation domain TAD2 of Ino2 (lanes 4), while TAD1 could not bind to Bdf1 (lanes 3). To exclude indirect interaction possibly mediated by other subunits of TFIID, we also produced HA-Bdf1 in *E. coli* and repeated the pull-down experiment. Indeed, binding of Ino2 (TAD2) to Bdf1 was confirmed with bacterially synthesized proteins, arguing for a direct interaction.

**Fig. 2:**
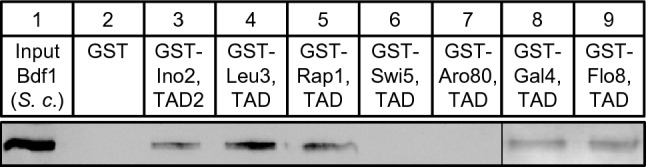
TADs of various unrelated transcriptional activators interact with Bdf1. GST fusions of Ino2 TAD2 (aa 101–135; pSH118), Leu3 TAD (aa 841–886; pES5), Rap1 TAD (aa 630–671; pLJ6), Swi5 TAD (aa 1–85; pDG1), Aro80 TAD (aa 846–950; pMG50), Gal4 TAD (aa 768–881; pES20) and Flo8 TAD (aa 726–799; pMG123) were synthesized in *E. coli*, immobilized on GSH sepharose and incubated with protein extract containing epitope-tagged full-length Bdf1 (yeast expression plasmid pJL84). Unfused GST served as a negative control

Since Bdf1 has not yet been identified as a target of activator proteins, we extended our interaction studies and investigated whether GST fusions of additional TADs can also bind to Bdf1. For these experiments, we used GST fusions of TADs taken from Leu3 (aa 841–886), Rap1 (aa 630–671; Johnson and Weil 2017), Swi5 (aa 1–85), Aro80 (aa 846–950), Gal4 (aa 768–881) and Flo8 (aa 726–799). These TAD sequences efficiently activated a *GAL1-lacZ* reporter gene when fused with the DNA-binding domain of Gal4 (data not shown). Our interaction experiments revealed that not only TAD2 of Ino2 (lane 3) but also activation domains of Leu3, Rap1, Gal4 and Flo8 (lanes 4, 5, 8 and 9) could bind to Bfd1, while no binding was detected with Swi5 and Aro80 (lanes 6 and 7; Fig. 2). We conclude that Bdf1 is a common target of activation domains from regulatory proteins of unrelated function.

**Fig. 3:**
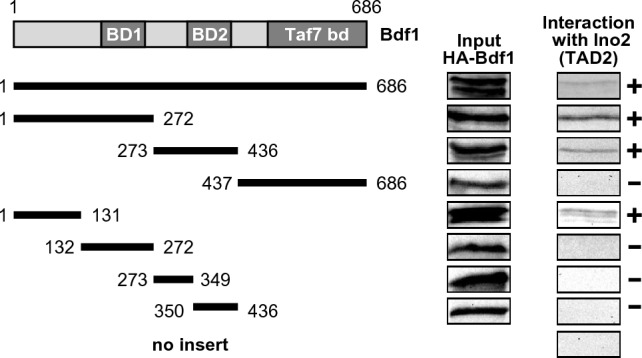
Mapping of Bdf1 domains interacting with TAD2 of Ino2. Fusion protein GST–Ino2 TAD2 (aa 101–135; pSH118) was synthesized in *E. coli*, immobilized on GSH sepharose and incubated with bacterial protein extracts containing epitope-tagged length variants of Bdf1. The following expression plasmids were used to synthesize HA-Bdf1 variants in *E. coli*: pMS161 (aa 1–686, full-length), pMS176 (aa 1–272), pMS177 (aa 273–436), pMS178 (aa 437–686), pMS189 (aa 1–131), pMS190 (aa 132–272), pMS191 (aa 273–349) and pMS192 (aa 350–436). As a negative control, empty GST plasmid was used (pGEX-2TK)

We next investigated whether interaction with TAD2 of Ino2 can be mapped to a defined region of the 686 aa long Bdf1 protein, containing two bromodomains (BD1, aa 169–255 and BD2, aa 338–422, respectively) and a C-terminal domain required for binding to Taf7 (aa 494–686; Matangkasombut et al. 2000). As is shown in Fig. 3, bacterially synthesized length variants aa 1–272 (containing BD1) and aa 273–436 (containing BD2) were able to interact with TAD2 of Ino2, while no interaction was detected with the Taf7-binding domain (aa 437–686). These results indicate that Bdf1 contains two non-overlapping activator-binding domains (ABD1 and ABD2). Further truncation of Bdf1 variant aa 1–272 showed that aa 1–131 also bound to Ino2, while aa 132–272 containing BD1 did not. We could no longer detect interaction with Ino2 when ABD2 was separated into shorter segments aa 273–349 and aa 350–436 (containing BD2), indicating that functionally important sequences of ABD2 were removed.

For a more precise analysis of ABD1, we next introduced mutations at selected positions into length variant aa 1–131, focusing on combinations of basic and hydrophobic residues conserved among various *Saccharomyces* yeasts (cf. sequence comparison as shown in Supplementary Fig. S1). Such residues were shown to be important for TAD binding in our analyses of ABDs in Taf1 and Taf12 (Hintze et al. 2017). Selected amino acids within Bdf1 aa 1–131 were replaced by alanine (generating double and triple mutants N17A V18A N19A, L26A K27A, L52A K53A K54A, E84A N85A and L101A K102A K103A) and subsequently used for in vitro interaction studies. Although in vitro interaction of Bdf1 aa 1–131 variant L52A K53A K54A was slightly less efficient than the wild type, the remaining four mutant variants interacted with Ino2 TAD2 similar as the wild type (Supplementary Fig. S2). We conclude that other positions within the ABD are required for contacting TAD2 or functional redundancies are effective among the selected residues (e.g., motifs LKK at aa 52–54 and aa 101–103).

### TFIIA subunit Toa1 interacts with both Ino2 activation domains

**Fig. 4:**
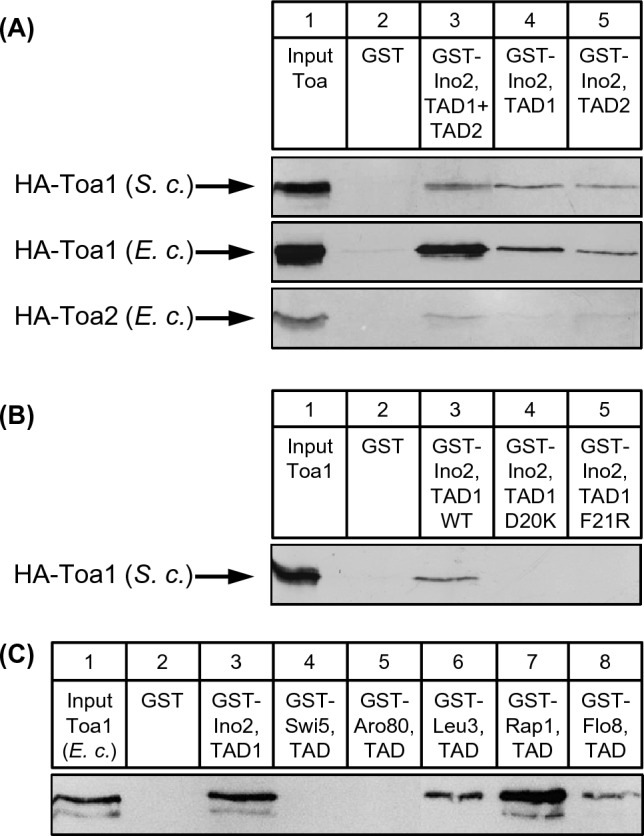
In vitro interaction assays (GST pull-down) with Ino2 activation domains and TFIIA subunits Toa1 and Toa2. **A** Fusion proteins GST–Ino2 (comprising TAD1 + TAD2, aa 1–135; encoded by pWTH12), GST–Ino2 TAD1 (aa 1–35; encoded by pSH117) and GST–Ino2 TAD2 (aa 101–135; pSH 118) were synthesized in *E. coli*, immobilized on GSH sepharose and incubated with protein extracts from transformants of *S. cerevisiae* (yeast expression plasmid pSH153; *MET25*-HA_3_-*TOA1*) or *E. coli* (expression plasmids pMS67, HA_3_-*TOA1*; and pMS76, HA_3_-*TOA2*). Negative control experiments were performed with GST (pGEX-2TK). **B** Interaction assays with wild-type and mutant variants of Ino2 TAD1, using GST fusion plasmids pSH117 (TAD1 wild-type), pSH122 (TAD1 D20K) and pSH123 (TAD1 F21R), respectively. HA-Toa1 was synthesized by yeast transformants containing pSH153. **C** Interaction assays with GST fusions of activation domains from Ino2 (TAD1, aa 1–35; pSH117), Swi5 (aa 1–85; pDG1), Aro80 (aa 846–950; pMG50), Leu3 (aa 841–886; pES5), Rap1 (aa 630–671; pLJ6) and Flo8 (aa 726–799; pMG123). HA-Toa1 was synthesized in *E. coli* (expression plasmid pMS67)

TFIIA enters the pre-initiation complex by binding to TFIID and stabilizes promoter binding of TFIID (Auty et al. 2004). In addition, TFIIA also functions as a coactivator and supports transcriptional activation by Rap1 (Papai et al. 2010) and Yap1 (Kraemer et al. 2006). To investigate its possible importance for activation by Ino2, we constructed expression plasmids to synthesize epitope-tagged variants of TFIIA subunits (HA-Toa1 and HA-Toa2) both in yeast and bacteria. For unknown reasons, we were unable to detect HA-Toa2 in yeast extracts and instead used bacterially synthesized HA-Toa2. For subsequent interaction studies, GST–Ino2 fusions comprising TAD1 + TAD2 as well as individual TADs were incubated with protein extracts from *S. cerevisiae* (HA-Toa1) or *E. coli* (HA-Toa1 and HA-Toa2). Results depicted in Fig. 4A show that Toa1 from either source could bind to TAD1 as well as to TAD2 (lanes 3, 4 and 5). Missense variants D20K and F21R of TAD1 being defective for transcriptional activation (Dietz et al. 2003) could no longer interact with HA-Toa1 synthesized in yeast (Fig. 4B, lanes 4 and 5). Toa2 may also interact with Ino2 but apparently substantially less efficient than Toa1. We thus did not perform additional experiments with the small Toa2 protein (122 aa) and instead concentrated on Toa1 (286 aa) for more precise mapping studies. To compare Ino2 with interaction specificities of other activators, we again used GST–TAD fusions from Swi5, Aro80, Leu3, Rap1 and Flo8 and studied their binding to Toa1 (Fig. 4C). These experiments showed that Leu3, Rap1 and Flo8 are additional activators which may use Toa1 for stimulation of gene expression (lanes 6, 7 and 8).

**Fig. 5:**
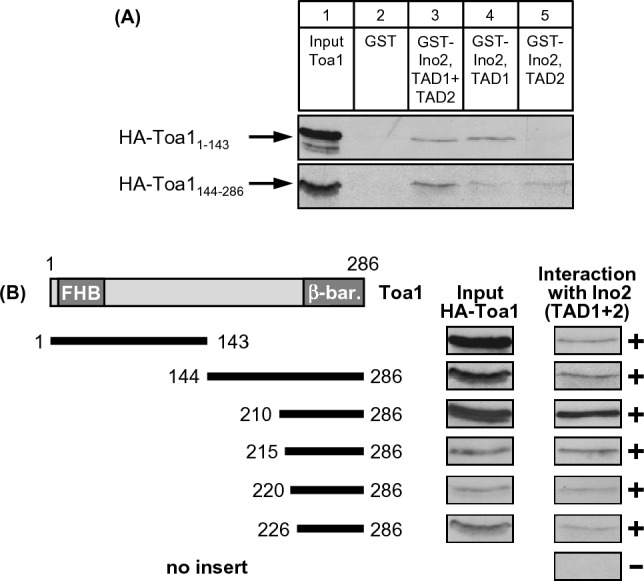
Mapping studies for identification of TAD-binding domains within Toa1. **A** GST fusion proteins representing TAD1 + TAD2 of Ino2 (aa 1–135; pWTH12) as well as individual TAD1 (aa 1–35; pSH117) and TAD2 (aa 101–135; pSH118) were immobilized on GSH sepharose and incubated with *E. coli* protein extracts containing epitope-tagged N- and C-terminal half of Toa1 (expression plasmids pMS69, HA_3_-*TOA1* encoding aa 1–143, and pMS70, HA_3_-*TOA1*, aa 144–286). **B** GST–Ino2 fusion protein containing TAD1 + TAD2 (aa 1–135; pWTH12) was incubated with bacterial protein extracts containing HA-labeled length variants of Toa1, using expression plasmids pMS69 (aa 1–143), pMS70 (aa 144–286), pMS71 (aa 210–286), pMS72 (aa 215–286), pMS73 (aa 220–286) and pMS74 (aa 226–286), respectively

Structural studies of yeast TFIIA by X-ray crystallography has revealed the existence of two α-helices at the N-terminus of Toa1 (similar to subunit α of human TFIIA) and three β-sheets at its C-terminus (similar to human subunit β) both of which are required for interaction with corresponding structures of Toa2 (human subunit γ), thus forming a four-helix-bundle (FHB) and a β-barrel (Geiger et al. 1996; Tan et al. 1996). In contrast to these structural motifs, the internal sequence of Toa1 (aa 51–220) does not show similarities to TFIIA subunits of *Drosophila* and human. Results of in vitro interaction studies showed that non-overlapping sequences of Toa1 at its N- and C-terminus could bind to TADs of Ino2 (Fig. 5A; lanes 3, 4 and 5). While the C-terminus of Toa1 containing its β-sheets interacted with TAD1 and TAD2, the N-terminus with its α-helices only bound to TAD1. Because of problems with the stable synthesis of more truncated length variants of the Toa1 N-terminus, we were unable to map its activator-binding domain (ABD1) more precisely. In contrast, length variants of the Toa1 C-terminus allowed us to map an activator-binding domain (ABD2) which co-localizes with its β-sheet structures (aa 226–286; Fig. 5B).

**Fig. 6:**
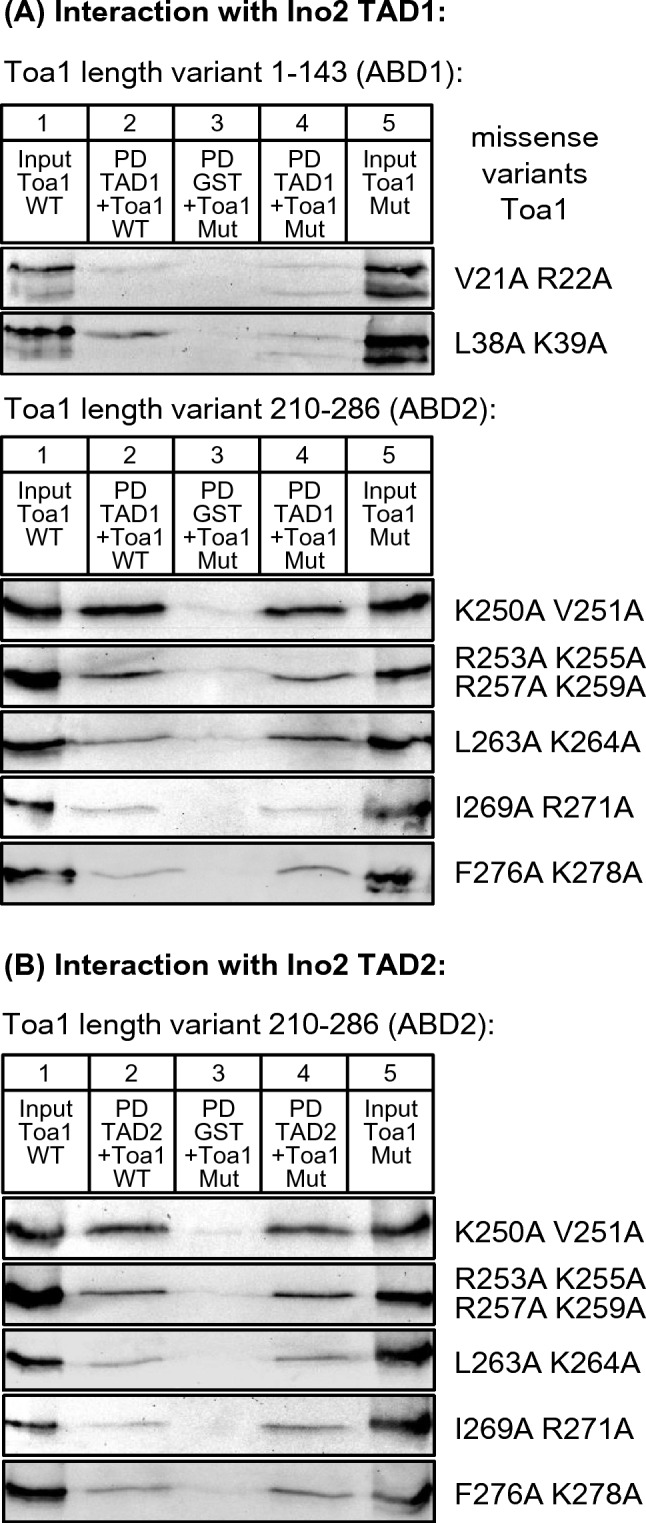
Comparative investigation of TAD–Toa1 interaction using missense variants within ABD1 and ABD2. **A** Interaction of Ino2 TAD1 with Toa1. Fusion protein GST–Ino2 TAD1 (pSH117) was incubated with bacterial protein extracts containing wild-type and mutant Toa1 ABD1 (aa 1–143, upper panel) and ABD2 (aa 210–286, lower panel), respectively. Input samples are shown in lanes 1 (wild-type, WT) and lanes 5 (mutant variant, Mut). Pull-down (PD) experiments were analyzed in lanes 2 (wild-type) and lanes 4 (mutant variant). Incubation of GST with mutant Toa1 variants served as negative control experiments (lanes 3). To synthesize epitope-tagged ABD1 of Toa1, expression plasmids pMS69 (encoding wild-type aa 1–143), pMS181 (V21A R22A) and pMS183 (L38A K39A) were used. Similarly, ABD2 was synthesized using plasmids pMS71 (encoding wild-type aa 210–286), pMS209 (K250A V251A), pMS210 (R253A K255A R257A K259A), pMS211 (L263A K264A), pMS212 (I269A R271A) and pMS213 (F276A K278A). **B** Interaction of Ino2 TAD2 with Toa1. Fusion protein GST–Ino2 TAD2 (pSH118) was incubated with bacterial protein extracts containing wild-type and mutant Toa1 ABD2 (210–286). Since Ino2 TAD2 is unable to interact with ABD1, variants V21A R22A and L38A K39A were assayed only for binding to TAD1

Since Toa1 length variants aa 1–143 and aa 210–286 could be efficiently synthesized in *E. coli* and showed intensive interaction with TADs of Ino2, we next wished to investigate the possible importance of selected amino acids for activator binding. Similar to mutational analysis of Bdf1, we selected combinations of hydrophobic and basic amino acids strongly conserved among *Saccharomyces* yeasts (V21 R22; L38 K39; K250 V251; L263 K264; I269 R271 and F276 K287, together with the basic cluster R253 K255 R257 K259; cf. sequence comparisons shown in Supplementary Fig. S3) and constructed missense variants by site-directed mutagenesis in which the mentioned amino acids were replaced by alanine residues. Five variants of Toa1 ABD2 (aa 210–286) were then used for comparative qualitative interaction studies using GST–Ino2 TAD1 and GST–Ino2 TAD2, respectively. Since ABD1 had turned out to be specific for TAD1 (Fig. 5A), two variants of Toa1 ABD1 (aa 1–143) were only investigated for binding to GST–Ino2 TAD1. As is apparent from Fig. 6A (interaction of TAD1 with Toa1 variants) and Fig. 6B (interaction of TAD2 with Toa1 variants), most of these variants were not significantly impaired for TAD binding. Only interaction of variant Toa1 ABD1 L38A R39A with TAD1 was less effective than wild-type ABD1. We conclude that TAD–Toa1 interaction may be functionally redundant or is dependent on other residues not considered for this mutagenesis.

**Fig. 7:**
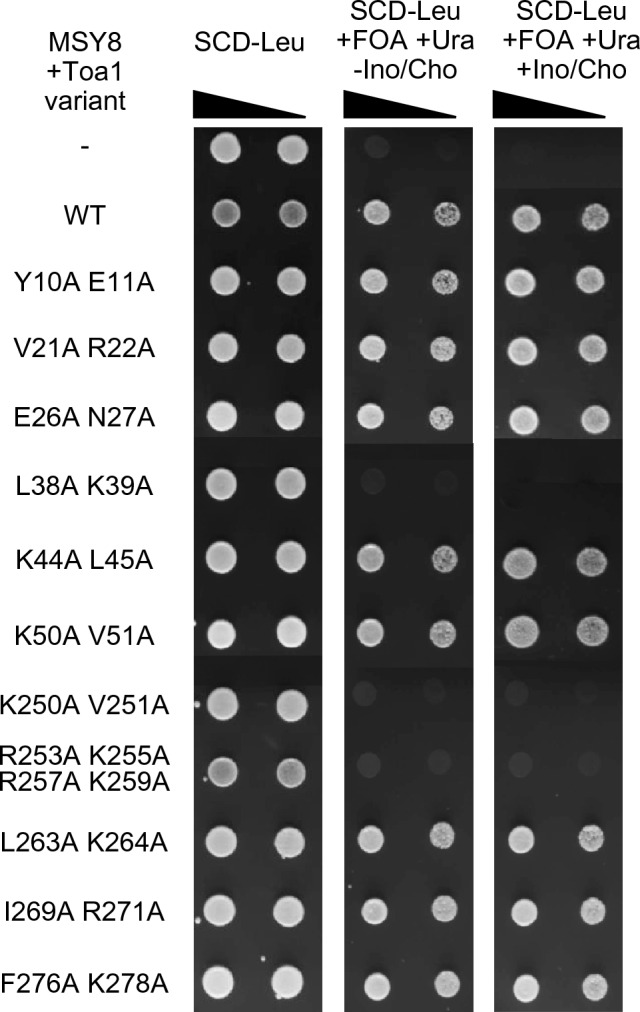
Functional analysis of Toa1 missense variants by plasmid shuffling experiments. Strain MSY8 contains a complete deletion of the chromosomal *TOA1* coding region and stays viable, because the *ARS CEN URA3 TOA1* rescue plasmid pMS114 is present. MSY8 was transformed with *ARS CEN LEU2 TOA1* plasmids containing the missense variants shown and selected for growth on synthetic medium containing 5-fluoroorotic acid (FOA), allowing counter-selection against pMS114. Growth on FOA-containing media was tested in the presence and in the absence of phospholipid precursor inositol + choline (Ino/Cho)

To investigate whether missense mutations affecting ABD1 or ABD2 functionally compromise full-length Toa1 in vivo, we next performed complementation studies using the plasmid shuffle strategy. We thus introduced a centromeric *URA3* rescue plasmid containing an intact *TOA1* gene into a regulatory wild-type strain and subsequently deleted the chromosomal *TOA1* copy, giving strain MSY8. Centromeric *LEU2* plasmids containing 11 *TOA1* variants with alterations of ABD1 or ABD2 were then transformed into MSY8. Cultivation in the presence of FOA selected for loss of the *URA3 TOA1* rescue plasmid and allowed us to assay whether *TOA1* variants could complement the chromosomal *toa1* null mutation. As is apparent from Fig. 7, eight Toa1 variants (Y10A E11A, V21A R22A, E26A N27A, K44A L45A, K50A V51A, L263A K264A, I269A R271A and F276A K278A) were able to fully replace wild-type Toa1, irrespective whether inositol and choline were available or not. *TOA1* variants mediating viability were also assayed for a possible influence on expression of an *INO1-lacZ* reporter gene, but β-galactosidase activities in extracts of yeast transformants were not significantly different from the wild-type control (data not shown). In contrast, three variants (L38A K39A, K250A V251A and R253A K255A R257A K259A) failed to complement the *toa1* null mutation, but this deficiency was not rescued by supplementation with inositol and choline. Presumably, deficiency of transcriptional activation may not be the reason why the basic cluster R253 K255 R257 K259 is functional essential. Instead, these amino acids have been shown to fulfill a general function for promoter recognition as they form ionic interactions or hydrogen bonds with DNA close to or within the TATA element (Geiger et al. 1996; Tan et al. 1996). Interestingly, the functional loss of variant ABD1 L38A K39A in vivo (Fig. 7) coincides with its reduced TAD interaction in vitro (Fig. 6), indicating that L38 K39 within the α-helical structure of Toa1 are not only involved in formation of the FHB dimerization surface with Toa2 but may also fulfill a coactivator function.

## Discussion

The various functions of mammalian TFIID subunit Taf1 are allocated on two separate proteins of yeast with Taf1 as the core domain being responsible for binding of basal promoter elements (Chalkley and Verrijzer 1999; Curran et al. 2018) and transcriptional activators (Lively et al. 2001; Hintze et al. 2017), while bromodomain-containing yeast proteins Bdf1/Bdf2 correspond to the “missing” C-terminal part of mammalian Taf1 and are preferentially required for recognition of acetyl lysine in histone H4 (Jacobson et al. 2000; Pamblanco et al. 2001). In previous work, we could show that yeast Taf1 contains two non-overlapping activator-binding domains (ABD) in its N-terminus which can interact with activation domains identified in Ino2, Adr1, Mac1, Met4, Pdc2 (Hintze et al. 2017; M. Grigat and HJS, unpublished). Bdf1 and Bdf2 have been identified as interaction partners of Taf7 and were initially considered as complementing factors which support binding of TFIID to epigenetic marks of the + 1 nucleosome within the basal promoter (Matangkasombut et al. 2000). In this work, we were able to demonstrate that Bdf1 (but not Bdf2) exerts an additional function, namely its ability to interact with activation domain TAD2 of Ino2, while its more efficient TAD1 was not bound by Bdf1. We could also show that unrelated activators Gal4, Rap1, Flo8 and Leu3 could bind to Bdf1 as well (proteome-wide investigations for proteins being able to physically interact with Bdf1 additionally identified activators Gcr1, Hot1 and Tea1; yeastgenome.org, contacted in June 2023). Importantly, Bdf1 and Bdf2 are not completely redundant as the mutant phenotype of *bdf1*Δ (deficiency to utilize nonfermentable carbon sources, among others) is much more distinct compared with *bdf2*Δ (no deficiencies; Matangkasombut et al. 2000). Recruitment of Bdf1 by activator proteins may provide an explanation why loss of *BDF1* correlates with a more severe phenotype than loss of *BDF2*.

**Fig. 8:**
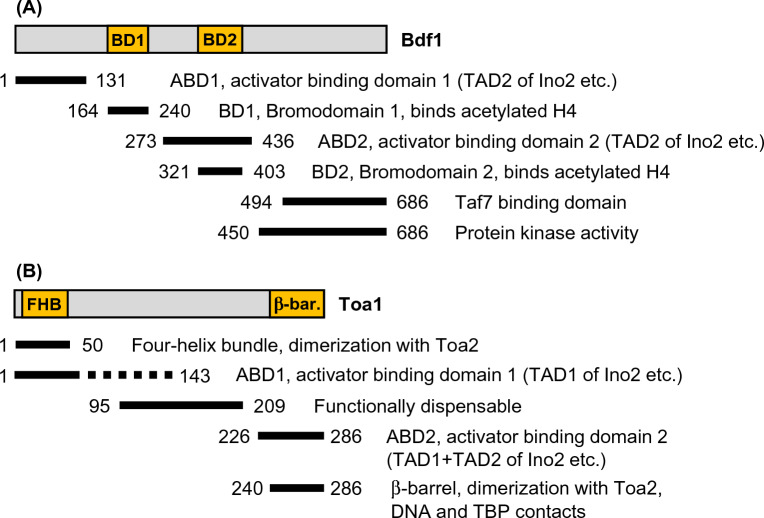
Functional domains of Bdf1 (**A**) and Toa1 (**B**). Data for domains within Bdf1 were taken from Matangkasombut et al. (2000), Matangkasombut and Buratowski (2003) and this work. Domains of Toa1 were structurally analyzed by Geiger et al. (1996) and Tan et al. (1996). Activator-binding domains were characterized in this work

Construction of Bdf1 length variants allowed us to identify two non-overlapping activator-binding domains (ABD1, aa 1–131 and ABD2, aa 273–436, respectively) both of which could interact with Ino2 TAD2 in experiments fully dependent on bacterially synthesized proteins, indicating a direct interaction which does not require yeast-specific proteins. While Bdf1 ABD1 was mapped within a protein sequence of yet undefined function, Bdf1 ABD2 overlaps with bromodomain BD2. However, mutation of selected combinations of basic-hydrophobic amino acids within ABD1 did not substantially abolish binding to Ino2 TAD2. Our results refine the molecular anatomy of Bdf1 which is summarized in Fig. 8A.

In addition to its function as a factor supporting and stabilizing interaction of TFIID with basal promoter elements, TFIIA also mediates the influence of transcriptional activator proteins in yeast, mammalian and viral systems (Kobayashi et al. 1995; Ozer et al. 1996). Previous work concentrated on the coactivator function of the small TFIIA subunit γ which is functionally interchangeable with Toa2 (Ozer et al. 1994) and interacts with activation domains of viral Zta and VP16 as well as with yeast activator Yap1 mediating response to oxidative stress. Mutational studies of Toa2 revealed that hydrophobic residues mapping within its FHB are important for interaction with Yap1, in vivo and in vitro (Kraemer et al. 2006). Although there is general agreement that immediate–early transcriptional activator Zta of Epstein–Barr virus interacts with TFIIA, different results were reported whether interaction is mediated by mammalian αβ or γ (similar to Toa1 and Toa2, respectively; Kobayashi et al. 1995; Ozer et al. 1994). In this work we demonstrate that both activation domains of Ino2 efficiently bind to Toa1, while only weak binding was observed with Toa2. We thus focused our analysis on Toa1 and could subsequently show that its interaction with Ino2 TAD1 was completely abolished with activation-defective TAD1 variants. These results agree with the previous finding that induced depletion of Toa1 led to substantially decreased transcription of *INO1* while genes of amino acid biosynthesis were only partially affected and *CUP1* was unaffected (Liu et al. 1999). Functionally unrelated yeast activators Rap1, Leu3 and Flo8 (but not Swi5 and Aro80) also bound to Toa1. Since our work concentrated on in vitro experiments, it remains open whether the interactions shown are also important for gene activation in vivo. Since TADs may contact a large number of coactivators, functional redundancy among them cannot be ruled out. Chromatin immunoprecipitation studies using yeast strains which synthesize epitope-tagged variants of Bdf1 and Toa1 could further support the findings reported in this work.

The crystal structure of TFIIA/TBP/TATA box shows that the four-helix bundle domain formed by Toa1 and Toa2 is in distance to TBP/TATA and thus not involved in DNA contacts with basal promoter sequences (Geiger et al. 1996; Tan et al. 1996). These results instead supported the view that dimerization with Toa2 and subsequent formation of the four-helix bundle may be the sole function of both α-helical regions within Toa1. Here, we demonstrate that a corresponding Toa1 length variant also mediates interaction with transcriptional activation domains which agrees with its position projecting away from TBP and TATA sequences. Introduction of the L38A K39A missense variant into the N-terminal Toa1 FHB domain led to a partial loss of interaction with Ino2 TAD1 and failure to complement a *toa1* null mutation. This finding is in accordance with growth deficiencies described for the related mutant variant L38A K39A N40A (Layer and Weil 2013).

Since the C-terminal β-barrel domain of Toa1 fulfills multiple functions and is in close contact to Toa2, TBP and DNA around the TATA element, we were surprised to find that this region also mediates activator contacts. Since we were unsuccessful to bacterially synthesize the core β-barrel domain, we cannot exclude that interaction also depends on additional sequences. Double mutation K250A V251A and quadruple mutation R253A K255A R257A K259A mapping within the Toa1 β-barrel domain completely failed to complement a *toa1* null mutation which is explained by loss of hydrogen-bond contacts within or close to the TATA element of the basal promoter (Geiger et al. 1996; Tan et al. 1996). However, activator binding was unaffected by both variants. A comprehensive mutational analysis of *TOA1* by small internal deletions and alanine scanning mutagenesis has been previously reported by Kang et al. (1995), demonstrating that most point mutations failed to cause a functional deficiency. In summary, our investigations contribute to a more complete picture of the functional diversity of TFIIA subunits and allow mapping of two separate activator-binding domains within Toa1 (ABD1 and ABD2; cf. Fig. 8B).

### Supplementary Information

Below is the link to the electronic supplementary material.

Supplementary file1 (DOCX 272 KB)

## Data Availability

Original data are available upon request. Additional information is provided in the Supplementary Material.
