# Supplementary material for: Ino2, activator of yeast phospholipid biosynthetic genes, interacts with basal transcription factors TFIIA and Bdf1
Source: Curr Genet. 2023 Nov 10;69(4-6):289–300. doi: 10.1007/s00294-023-01277-z (PMC10716077; doi:10.1007/s00294-023-01277-z)
Supplement: Supplementary file 1 — Supplementary file1 (DOCX 272 KB) [file 294_2023_1277_MOESM1_ESM.docx]

**Supplementary Online Material Engelhardt et al.:**

**Table S1: Strains of *Saccharomyces cerevisiae*:**

| **Strain** | **Genotype** |
| --- | --- |
| C13-ABY.S86 | *MAT*α *ura3 leu2 pra1 prb1 prc1 cps1* |
| MSY8 | *MAT*α *ura3 leu2 trp1 his3 toa1*Δ*::loxP* [*ARS CEN URA3 TOA1*] |

**Table S2: Plasmids constructed and used:**

| **Plasmid** | **Genotype** |
| --- | --- |
| pDG1 | tac_Pr/Op_-*GST-SWI5*_1-85_ |
| pECW59 | tet_Pr/Op_-HA_3_-*BDF1*_1-131_ N17A V18A N19A |
| pECW60 | tet_Pr/Op_-HA_3_-*BDF1*_1-131_ L26A K27A |
| pECW61 | tet_Pr/Op_-HA_3_-*BDF1*_1-131_ L52A K53A K54A |
| pES20 | tac_Pr/Op_-*GST-GAL4*_768-881_ |
| pES5 | tac_Pr/Op_-*GST-LEU3*_841-886_ |
| pJL84 | 2µm *LEU2 MET25*_Pr_-HA_3_-*BDF1* |
| pJL85 | 2µm *LEU2 MET25*_Pr_-HA_3_-*BDF2* |
| pLJ6 | tac_Pr/Op_-*GST-RAP1*_630-671_ |
| pMG123 | tac_Pr/Op_-*GST-FLO8*_726-799_ |
| pMG50 | tac_Pr/Op_-*GST-ARO80*_846-950_ |
| pMS67 | tet_Pr/Op_-HA_3_-*TOA1* |
| pMS69 | tet_Pr/Op_-HA_3_-*TOA1*_1-143_ |
| pMS70 | tet_Pr/Op_-HA_3_-*TOA1*_144-286_ |
| pMS71 | tet_Pr/Op_-HA_3_-*TOA1*_210-286_ |
| pMS72 | tet_Pr/Op_-HA_3_-*TOA1*_215-286_ |
| pMS73 | tet_Pr/Op_-HA_3_-*TOA1*_220-286_ |
| pMS74 | tet_Pr/Op_-HA_3_-*TOA1*_226-286_ |
| pMS76 | tet_Pr/Op_-HA_3_-*TOA2* |
| pMS113 | *toa1*Δ::*LEU2* |
| pMS114 | *ARS CEN URA3 TOA1* (vector: pRS416) |
| pMS115 | *ARS CEN LEU2 TOA1* (vector: YCplac111) |
| pMS147 | *ARS CEN LEU2 TOA1* K250A V251A |
| pMS148 | *ARS CEN LEU2 TOA1* R253A K255A R257A K259A |
| pMS149 | *ARS CEN LEU2 TOA1* L263A K264A |
| pMS150 | *ARS CEN LEU2 TOA1* I269A R271A |
| pMS151 | *ARS CEN LEU2 TOA1* F276A K278A |
| pMS161 | tet_Pr/Op_-HA_3_-*BDF1* |
| pMS176 | tet_Pr/Op_-HA_3_-*BDF1*_1-272_ |
| pMS177 | tet_Pr/Op_-HA_3_-*BDF1*_273-436_ |
| pMS178 | tet_Pr/Op_-HA_3_-*BDF1*_437-686_ |
| pMS181 | tet_Pr/Op_-HA_3_-*TOA1*_1-143_ V21A R22A |
| pMS183 | tet_Pr/Op_-HA_3_-*TOA1*_1-143_ L38A K39A |
| pMS189 | tet_Pr/Op_-HA_3_-*BDF1*_1-131_ |
| pMS190 | tet_Pr/Op_-HA_3_-*BDF1*_132-272_ |
| pMS191 | tet_Pr/Op_-HA_3_-*BDF1*_273-349_ |
| pMS192 | tet_Pr/Op_-HA_3_-*BDF1*_350-436_ |
| pMS203 | *ARS CEN LEU2 TOA1* Y10A E11A |
| pMS204 | *ARS CEN LEU2 TOA1* V21A R22A |
| pMS205 | *ARS CEN LEU2 TOA1* E26A N27A |
| pMS206 | *ARS CEN LEU2 TOA1* L38A K39A |
| pMS207 | *ARS CEN LEU2 TOA1* K44A L45A |
| pMS208 | *ARS CEN LEU2 TOA1* K50A V51A |
| pMS209 | tet_Pr/Op_-HA_3_-*TOA1*_210-286_ K250A V251A |
| pMS210 | tet_Pr/Op_-HA_3_-*TOA1*_210-286_ R253A K255A R257A K259A |
| pMS211 | tet_Pr/Op_-HA_3_-*TOA1*_210-286_ L263A K264A |
| pMS212 | tet_Pr/Op_-HA_3_-*TOA1*_210-286_ I269A R271A |
| pMS213 | tet_Pr/Op_-HA_3_-*TOA1*_210-286_ F276A K278A |
| pMS222 | tet_Pr/Op_-HA_3_-*BDF1*_1-131_ L101A K102A K103A |
| pMS223 | tet_Pr/Op_-HA_3_-*BDF1*_1-131_ E84A N85A |
| pSH62 | 2µm *HIS3 GAL1-cre* |
| pSH117 | tac_Pr/Op_-*GST-INO2*_1-35_ (TAD1); Hintze et al. 2017 |
| pSH118 | tac_Pr/Op_-*GST-INO2*_101-135_ (TAD2); Hintze et al. 2017 |
| pSH122 | tac_Pr/Op_-*GST-INO2*_1-35_ D20K (mutTAD1); Hintze et al. 2017 |
| pSH123 | tac_Pr/Op_-*GST-INO2*_1-35_ F21R (mutTAD1); Hintze et al. 2017 |
| pSH153 | 2µm *URA3 MET25*_Pr_-HA_3_-*TOA1* |
| pWTH12 | tac_Pr/Op_-*GST-INO2*_1-135_ (TAD1+TAD2); Hintze et al. 2017 |

Pr, promoter; Op, operator.

**Table S3: Oligonucleotides used (PCR primers for construction of *BDF1* and *TOA1* length variants, site-directed mutagenesis):**

| **Name** | **Gene** | **Position** | **Sequence 5‘-3‘** |
| --- | --- | --- | --- |
| Aro80 Bam Start 846 | *ARO80* | +2536/  +2555 | gatc**ggatcc**TCTCAGAATTCAAATGATAC |
| Aro80 Pst Stop 950 | *ARO80* | +2853/  +2834 | gatc**ctgcag**TTATTTACGCGTTATTGGCC |
| Flo8 BamHI Start 726 | *FLO8* | +2176/  +2197 | gatc**ggatcc**TCTACAAATAGTGGCGATAATG |
| Flo8 SalI Stop 799 | *FLO8* | +2400/  +2378 | gatc**gtcgac**TCAGCCTTCCCAATTAATAAAAT |
| Gal4 BamHI Start 768 | *GAL4* | +2302/  +2322 | gatc**ggatcc**GCCAATTTTAATCAAAGTGGG |
| Gal4 HindIII Stop 881 | *GAL4* | +2646/  +2626 | gatc**aagctt**TTACTCTTTTTTTGGGTTTGG |
| Leu3 BamHI Start 841 | *LEU3* | +2521/  +2541 | gatc**ggatcc**GTTCAATCAAACCCCGTTAC |
| Leu3 SalI Stop 886 | *LEU3* | +2658/  +2637 | gatc**gtcgac**TTAAACCTTGGGATTGAACGC |
| Rap1 Start 630 BamHI | *RAP1* | +1888/  +1910 | gatc**ggatcc**TCTTACGCTATACCAGAAAACG |
| Rap1 Stop 671 Sall | *RAP1* | +2181/  +2159 | gatc**gtcgac**CTATTCCGCAATCTCGTGTGGAT |
| Swi5 BamHI Stop 85 | *SWI5* | +255/+236 | gatc**ggatcc**TCAAGATTTTTCATCAGAAAGTG |
| Swi5 EcoRI Start 1 | *SWI5* | +1/+21 | gatc**gaattc**ATGGATACATCAAACTCTTGG |
| Bdf1 1-131 NVN-17 18 19-AAA 3R | *BDF1* | +72/+31 | ACTGGAAACGTCGTCAGCGGCAGCATTACCATTGACATCCAC |
| Bdf1 1-131 NVN-17 18 19-AAA 5F | *BDF1* | +31/+72 | GTGGATGTCAATGGTAATGCTGCCGCTGACGACGTTTCCAGT |
| Bdf1 1-131 LK-26 27-AA 3R | *BDF1* | +104/+58 | GGATCCCCTTGATCTATAGGCCTCGCTGCATTACTGGAAACGTCGTC |
| Bdf1 1-131 LK-26 27-AA 5F | *BDF1* | +58/+104 | GACGACGTTTCCAGTAATGCAGCGAGGCCTATAGATCAAGGGGATCC |
| Bdf1 1-131 LKK-52 53 54-AAA 3R | *BDF1* | +178/+134 | CTCCATCCAGTCTAGCCGCTGCGGCATGCAACTGGTTATTGGCGG |
| Bdf1 1-131 LKK-52 53 54-AAA 5F | *BDF1* | +134/+178 | CCGCCAATAACCAGTTGCATGCCGCA  GCGGCTAGACTGGATGGAG |
| Bdf1 1-131 EN-84 85-AA 3R | *BDF1* | +283/+237 | CTGCGCCACTTCCGGTGGCGTTATACCCAGCTGCCCCGTTAGCCGCC |
| Bdf1 1-131 EN-84 85-AA 5F | *BDF1* | +237/+283 | GGCGGCTAACGGGGCAGCTGGGTATAACGCCACCGGAAGTGGCGCAG |
| Bdf1 1-131 LKK-101 102 103-AAA 3R | *BDF1* | +325/+277 | CTTGTCCTCCTTCTTCCGCCGCCGCCCCCTGCTGTTCGTCTTCTGCGCC |
| Bdf1 1-131 LKK-101 102 103-AAA 5F | *BDF1* | +277/+325 | GGCGCAGAAGACGAACAGCAGGGGGCGGCGGCGGAAGAAGGAGGACAAG |
| Bdf1 PstI 5F | *BDF1* | +1/+19 | gatc**ctgcag**ATGACCGATATCACACCCG |
| Bdf1 aa 131 XhoI 3R | *BDF1* | +363/+346 | gatc**ctcgag**TCATTGTTTTGAGTTTTCATC |
| Bdf1 aa 132 PstI 5F | *BDF1* | +364/+383 | gatc**ctgcag**GAACTTCCGATGGAGGTTCC |
| Bdf1 aa 272 XhoI 3R | *BDF1* | +816/+801 | gatc**ctcgag**TCATTGGGCACTAGACCGC |
| Bdf1 aa 273 PstI 5F | *BDF1* | +817/+839 | gatc**ctgcag**GAGGATGCCCCAATTGTAATTAG |
| Bdf1 aa 349 XhoI 3R | *BDF1* | +1047/  +1030 | gatc**ctcgag**TCACATAGAAACTGGGTCTAC |
| Bdf1 aa 350 PstI 5F | *BDF1* | +1047/  +1067 | gatc**ctgcag**AATTTGCCGACTTATTTCGA |
| Bdf1 aa 436 XhoI 3R | *BDF1* | +1308/  +1291 | gatc**ctcgag**TCATTGGGTCCTCGAATCTTC |
| Bdf1 aa 437 PstI 5F | *BDF1* | +1309/  +1327 | gatc**ctgcag**GGCGACTACGACGATTATG |
| Bdf1 XhoI 3R | *BDF1* | +2061/  +2040 | gatc**ctcgag**TCACTCTTCTTCACTTTCGCTG |
| Bdf2 BamHI 5F | *BDF2* | +1/+20 | gatc**ggatcc**ATGTCTCGTACTAACATGGA |
| Bdf2 HindIII 3R | *BDF2* | +1917/  +1898 | gatc**aagctt**TTAATCACTGTCACTGTCGC |
| Toa1 BamHI 5F | *TOA1* | +1/+20 | gatc**ggatcc**ATGTCGAATGCAGAGGCCAG |
| Toa1 HindIII 3R | *TOA1* | +861/+842 | gatc**aagctt**TTATACCCACTCCGCTTCCA |
| Toa1 aa 143 HindIII 3R | *TOA1* | +429/+410 | gatc**aagctt**TCAGTCCGCATTTGTATTACCAC |
| Toa1 aa 144 BamHI 5F | *TOA1* | +430/+449 | gatc**ggatcc**GTAACTAGCCAGCCTAAAAT |
| Toa1 aa 210 BamHI 5F | *TOA1* | +628/647 | gatc**ggatcc**AGTGCATTATTAGATACGGA |
| Toa1 aa 215 BamHI 5F | *TOA1* | +643/+664 | gatc**ggatcc**ACGGATGAGGTCGGTTCAGAAC |
| Toa1 aa 220 BamHI 5F | *TOA1* | +658/+677 | gatc**ggatcc**TCAGAACTAGATGATTCCGA |
| Toa1 aa 226 BamHI 5F | *TOA1* | +673/+695 | gatc**ggatcc**GACGATGACTATCTAATTTC |
| Toa1 Y10A E11A 3R | *TOA1* | +61/+10 | CTTCATTTACCACAGACTCTACGATAATCGCGGCCACTCTGCTGGCCTCTGC |
| Toa1 Y10A E11A 5F | *TOA1* | +10/+61 | GCAGAGGCCAGCAGAGTGGCCGCGATTATCGTAGAGTCTGTGGTAAATGAAG |
| Toa1 V21A R22A 3R | *TOA1* | +86/+39 | CCCGCATTTTCAAAGTCCTCTGCTGCTTCATTTACCACAGACTCTACG |
| Toa1 V21A R22A 5F | *TOA1* | +39/+86 | CGTAGAGTCTGTGGTAAATGAAGCAGCAGAGGACTTTGAAAATGCGGG |
| Toa1 E26A N27A 3R | *TOA1* | +106/+58 | GTAAAGTTTGTTCATCGATACCCGCA  GCTGCAAAGTCCTCTCTTACTTC |
| Toa1 E26A N27A 5F | *TOA1* | +58/+106 | GAAGTAAGAGAGGACTTTGCAGCTGCGGGTATCGATGAACAAACTTTAC |
| Toa1 L38A K39A 3R | *TOA1* | +140/+90 | GTGAGCTTTTTTTGCCAAATATTTGCT  GCGTCTTGTAAAGTTTGTTCATCG |
| Toa1 L38A K39A 5F | *TOA1* | +90/+140 | CGATGAACAAACTTTACAAGACGCAGCAAATATTTGGCAAAAAAAGCTCAC |
| Toa1 K44A L45A 3R | *TOA1* | +158/+109 | GTAGTTACCTTCGTCTCTGTGGCCGCTTTTTGCCAAATATTTTTTAGGTC |
| Toa1 K44A L45A 5F | *TOA1* | +109/+158 | GACCTAAAAAATATTTGGCAAAAAGCGGCCACAGAGACGAAGGTAACTAC |
| Toa1 K50A V51A 3R | *TOA1* | +187/+135 | CTTCATTGAACTGATTGTCCCATGAAAAAGTAGTTGCCGCCGTCTCTGTGAGC |
| Toa1 K50A V51A 5F | *TOA1* | +135/+187 | GCTCACAGAGACGGCGGCAACTACTTTTTCATGGGACAATCAGTTCAATGAAG |
| Toa1 K250A V251A 3R | *TOA1* | +773/+718 | CATCTCGCCTTTGTTCTTGTGGCTGCATCATATAAGCACAGCATTAAGTTCTCATC |
| Toa1 K250A V251A 5F | *TOA1* | +718/+773 | GATGAGAACTTAATGCTGTGCTTATATGATGCAGCCACAAGAACAAAGGCGAGATG |
| Toa1 R253 K255 R257 K259-AAAA 3R | *TOA1* | +790/+738 | CTTTCAGACTACATGCCCATGCCGCC  GCTGTTGCTGTGACTTTATCATATAAG |
| Toa1 R253 K255 R257 K259-AAAA 5F | *TOA1* | +738/+790 | CTTATATGATAAAGTCACAGCAACAGCGGCGGCATGGGCATGTAGTCTGAAAG |
| Toa1 L263A K264A 3R | *TOA1* | +807/+758 | GATGGTCACCACACCATCTGCCGCACTACATTTCCATCTCGCCTTTGTTC |
| Toa1 L263A K264A 5F | *TOA1* | +758/+807 | GAACAAAGGCGAGATGGAAATGTAGT  GCGGCAGATGGTGTGGTGACCATC |
| Toa1 I269A R271A 3R | *TOA1* | +838/+790 | GAGCTTTTTGGAATGTGTAGTCATTTGCATTGGCGGTCACCACACCATC |
| Toa1 I269A R271A 5F | *TOA1* | +790/+838 | GATGGTGTGGTGACCGCCAATGCAAATGACTACACATTCCAAAAAGCTC |
| Toa1 F276A K278A 3R | *TOA1* | +854/+804 | CACTCCGCTTCCACTTGAGCTGCTTGGGCTGTGTAGTCATTTCTATTGATG |
| Toa1 F276A K278A 5F | *TOA1* | +804/+854 | CATCAATAGAAATGACTACACAGCCCAAGCAGCTCAAGTGGAAGCGGAGTG |
| Toa2 BamHI 5F | TOA2 | +1/+20 | gatc**ggatcc**ATGGCAGTACCCGGGTATTA |
| Toa2 HindIII 3R | TOA2 | +389/+348 | gatc**aagctt**TTACTCGCTCTTTTTTGAGTTA |

Artificially inserted cleavage sequences for restriction enzymes are shown in **bold**; capital letters represent genuine gene-specific sequences; capital letters underlined indicate sequences introducing site-specific mutations (GCN for alanine).

**Supplementary Figures:**

Scer 1 MTDITPVQNDVDVNGN**NVN**DDVSSN**LK**RPIDQ-----GDPSNGLAEEENP 45

Sbou 1 MTDITPVQNDVDVNGNNVNDDVSSNLKRPIDQ-----GDPSNGLAEEENP 45

Skud 1 MTDITPVQNDVDVNISNVNGDVSSNLKRHIDQVQGENGNSSNGLAEDKTP 50

Sarb 1 MTDISPVHNDVDVNGSNVNGDVSSNLKRSLDQVQDERGVFSNGLAEKKTP 50

Spar 1 MTDITPVQNDVDVNDSNVNGDVSSNLKRPMDQAQEVNGGSSNGLAEDETP 50

Seub 1 MTDITPVENDVDVN-GNVNGDISSTLKRPIDQI-------SNKDEGENGG 42

Scer 46 ANNQLH**LKK**ARLDGDALTSSP-----------AGLAENGI-EGATLAANG 83

Sbou 46 ANNQLHVKKARLDGDALTSAP-----------AGLAENGI-EGATLAANG 83

Skud 51 VNDELNTKKVKLNGGAFTTAP-----------VQLEENGTVEDSTLAANE 89

Sarb 51 VNDELDVKKARLDGDAFTSES-----------SQLAENGVVEGAKLAANG 89

Spar 51 ANDELHLKKARLNGNAPISTP-----------AQLAENGI-EGATLAANE 88

Seub 43 PLNGLAEDKTPINDETHTKKPKLNGDISSSASTQAAENGFVESPASAANE 93

Scer 84 **EN**GYNATGSGAEDE-QQG**LK**K---EEGGQGTKQEDLDENS--KQELPMEV 127

Sbou 84 ENGYNATGSGIEDE-QQGLKK---EEGGQGTKQEDLDENS--KQELPMEV 127

Skud 90 ENAYNATGVSAKEE-QQGLKKK--EEGEQGGEQGNFFSNEELKQEL---- 132

Sarb 90 ENAYNTTGVVTEEK-KQELKDEK-EEEHGGKQQSPV-GNEGLELEL---- 132

Spar 89 ENGYIATAGGAEKE-QHGLKKEGGEQGTKQEDADEDSKAK-----LPMEA 132

Seub 94 NDTYNATEVRAEEESQQVLKRENEEGHGGKQKSATANDGS--KLGLSEEA 142

Scer 128 PKE 130

Sbou 128 PKE 130

Skud 133 PKE 135

Sarb 133 PKE 135

Spar 133 PKE 135

Seub 143 PKE 145

**Supplementary Fig. S1:** Comparison of Bfd1 N-terminal sequences from various *Saccharomyces* yeasts. Scer, *S. cerevisiae*; Sbou, *S. boulardii*; Skud, *S. kudriavzevii*; Sarb, *S. arboricola*; Spar, *S. paradoxus*; Seub, *S. eubayanus*. Identical amino acids are indicated by grey shadowing; mutagenized residues within *S. cerevisiae* Bdf1 are shown in bold red letters.

**Supplementary Fig. S2:** Comparative investigation of TAD2-Bdf1 interaction using missense variants within Bdf1 1-131. GST fusion protein representing TAD2 of Ino2 (pSH118) was incubated with bacterial protein extracts containing epitope-tagged Bdf1 (plasmid pMS189, encoding wild-type aa 1-131) as well as variants N17A V18A N129A (pECW59), L26A K27A (pECW60), L52A K53A K54A (pECW61), E84A N85A (pMS222) and L101A K102A K103A (pMS223). Input samples are shown in lanes 1 (wild-type, WT) and lanes 5 (mutant variant, Mut). Pull-down (PD) experiments were analyzed in lanes 2 (wild-type) and lanes 4 (mutant variant).

(a) N-terminal Toa1 sequences (α-helical):

Scer 1 ----------------------------------MSNAEASRV**YE**IIVES 16

Smik 1 -----MAYYRGQAQKIKAIVVQRSDHREENSLDVMSNAEASRAYEIIVES 45

Spar 1 ----------------------------------MSNAEASRVYEIIVES 16

Suva 1 MDKAIRCTIGDKPRRKSDSRTGIEATQKGRSLDTMSNAEASRAYEIIVES 50

Sbay 1 ----------------------------------MSNAEASRAYEIIVES 16

Scas 1 ----------------------------------MSNPEASKVYELIVDS 16

Sklu 1 ----------------------------------MSNVEACRIYETVVES 16

Scer 17 VVNE**VR**EDF**EN**AGIDEQTLQD**LK**NIWQK**KL**TET**KV**TTFSWDNQF 60

Smik 46 VVNEVREDFENAGIDEQTLQDLKNIWQKKLTETKVSTFSWDNQF 89

Spar 17 VVNEVREDFENAGIDEQTLQDLKNIWQKKLTETKVTTFSWDNQF 60

Suva 51 VVNEVREDFENAGIDEQTLQDLKNIWQKKLTETKVTTFSWDNQF 94

Sbay 17 VVNEVREDFENAGIDEQTLQDLKNIWQKKLTETKVTTFSWDNQF 60

Scas 17 VINEVREDFENAGIDEQTLQDLKRVWQTKLTETKVTNFTWDDEL 60

Sklu 17 VVNEVREDFENAGIDEQTLQDLRRVWQSKLSDSGVCKFSWDPEP 60

(b) C-terminal Toa1 sequences (β-sheet):

Scer 215 TDEVGSELDDSDDDYLISEGEEDGPDENLMLCLYD**KV**T**R**T**K**A**R**W**K**CS**LK**D 264

Smik 239 TDEVGSELDDSDDDYLISEGEEDGPDENLMLCLYDKVTRTKARWKCSLKD 288

Spar 215 TDEVGSELDDSDDDYLISEGEEDGPDENLMLCLYDKVTRTKARWKCSLKD 264

Suva 243 TDEVGSELDDSDDDYLISEGEEDGPDENLMLCLYDKVTRTKARWKCSLKD 292

Sbay 209 TDEVGSELDDSDDDYLISEGEEDGPDENLMLCLYDKVTRTKARWKCSLKD 258

Scas 171 NDEVGSELDDSDDDYLISEGEDDGPDENLMLCLYDKVTRTKARWKCSLKD 220

Sklu 141 TDEINSDLDDSEDDYLNSSGDEETADENIVLCLYEKVLRVKNKWKCNLKD 190

Scer 265 GVVT**I**N**R**NDYT**F**Q**K**AQVEAEWV* 286

Smik 289 GVVTINRND* 297

Spar 265 GVVTINRNDYTFQKAQVEAEWV* 286

Suva 293 GVVTINRNDYTFQKAQVEAEWV* 314

Sbay 259 GVVTINRNDYTFQKAQVEAEWV* 280

Scas 221 GIVTINHKDYSFQKAQVEAEWV* 242

Sklu 191 GIATINHKDYAFQKAQGESEW* 211

**Supplementary Fig. S3:** Comparison of N-terminal (a) and C-terminal Toa1 sequences (b) from various *Saccharomyces* yeasts. Scer, *S. cerevisiae*; Smik, *S. mikatae*; Spar, *S. paradoxus*; Suva, *S. uvarum*; Sbay, *S. bayanus*; Scas, *S. castellii*; (*Naumovozyma castellii*); Sklu, *S. kluyveri* (*Lachancea kluyveri*). Identical amino acids are indicated by grey shadowing; mutagenized residues within *S. cerevisiae* Toa1 are shown in bold red letters.
